# Inequalities in US Life Expectancy by Area Unemployment Level, 1990–2010

**DOI:** 10.1155/2016/8290435

**Published:** 2016-03-17

**Authors:** Gopal K. Singh, Mohammad Siahpush

**Affiliations:** ^1^US Department of Health and Human Services, 5600 Fishers Lane, Rockville, MD 20857, USA; ^2^Department of Health Promotion, Social and Behavioral Health, University of Nebraska Medical Center, College of Public Health, Omaha, NE 68198-4365, USA

## Abstract

This study examined the association between unemployment and life expectancy in the United States during 1990–2010. Census-based unemployment rates were linked to US county-level mortality data. Life expectancies were calculated by age, sex, race, and unemployment level during 1990–2010. Differences in life expectancy were decomposed by age and cause of death. Life expectancy was consistently lower in areas with higher unemployment rates. In 2006–2010, those in areas with high unemployment rates (≥9%) had a life expectancy of 76.9 years, compared with 80.7 years for those in areas with low unemployment rates (<3%). The association between unemployment and life expectancy was stronger for men than for women. Life expectancy ranged from 69.9 years among black men in high unemployment areas to 90.0 years among Asian/Pacific Islander women in low unemployment areas. Disparities persisted over time. In 1990–1992, life expectancy was 4.7 years shorter in high unemployment than in low unemployment areas. In 2006–2010, the life expectancy difference between the lowest and highest unemployment areas decreased to 3.8 years. Heart disease, cancer, homicide, unintentional injuries, diabetes, HIV/AIDS, and liver cirrhosis contributed most to the lower life expectancy in high unemployment areas. High unemployment areas recorded larger gains in life expectancy than low unemployment areas, contributing to the narrowing gap during 1990–2010.

## 1. Introduction

Life expectancy is one of the most widely used indicators of health status and a key measure of human development [1, 2]. For more than two decades, increasing life expectancy and reducing health disparities among Americans have been the two most important health policy goals for the United States, as specified in its national health initiative,* Healthy People 2020* [1, 2].

Life expectancy estimates are routinely available for sex and broad racial/ethnic groups in the United States [3–5]. A few US studies have reported rural-urban and socioeconomic differentials in life expectancy, particularly by area-based poverty or deprivation level [2, 6–8]. Estimation of US life expectancy according to unemployment levels has received less attention, particularly the analysis of trends in life expectancy among areas or populations with high unemployment rates [7]. Although substantial disparities in life expectancy have been reported among sex, racial/ethnic, and socioeconomic groups in the USA, it is important to know the magnitude and causes of life expectancy disparities among populations with different unemployment rates for formulating comprehensive public policies to tackle the problem of growing health and socioeconomic inequalities in the country [6].

Studying the association of unemployment with life expectancy is important because unemployment is an important social determinant of health and is strongly linked to a number of health outcomes such as all-cause mortality, self-assessed health, cardiovascular diseases, liver cirrhosis, suicide, and mental health problems in a number of countries [9–12]. Besides its adverse effects on many health outcomes, unemployment is also related to smoking, obesity, heavy alcohol consumption, unhealthy diet, chronic stress, social isolation, and reduced health care access, all of which are associated with increased risks of morbidity and mortality [9–17]. Estimating health inequalities according to unemployment level has become especially important in light of the recent global economic downturn that saw US unemployment rates reach record levels at 9.6% during 2009-2010, with the current unemployment rates yet to reach precrisis levels of 2008 or before [10, 12, 18, 19].

Life expectancy is a summary measure of mortality that can be used to document both absolute and relative inequalities in survival between populations with low and high unemployment rates [2, 6]. It shows the effects of excess mortality risks during childhood, youth, working ages, and old age as well as those from major communicable and noncommunicable diseases and injuries [2]. The aim of our study is to estimate the magnitude of the association between unemployment level and life expectancy in the United States during 1990–2010. We link census-based unemployment rates to national mortality data to examine the extent to which life expectancy differences among US men and women in areas with varying unemployment levels have changed during the past two decades. We derive life expectancies at birth and other ages in low, medium, and high unemployment areas stratified by race/ethnicity and sex. We also decompose disparities in life expectancy attributable to excess mortality in specific age groups and causes of death.

## 2. Methods

The 1990–2010 national-vital-statistics-mortality database was used to analyze temporal unemployment inequalities in US life expectancy [2–6, 20, 21]. Since employment status of decedents is not reported in the national mortality database and direct computation of life expectancy by unemployment level is not possible, the 1990 and 2000 census-based unemployment data were linked to the age-sex-race-county-specific mortality statistics during 1990–1998 and 1999–2010, respectively, to derive life expectancy estimates [4, 12, 20–22].

Four unemployment rate categories were considered: <3.00% (i.e., less than 3.00% of the civilian labor force unemployed in a county, referred to as the low unemployment group), 3.00–5.99%, 6.00–8.99%, and 9.00% or higher (high unemployment group) [12]. Similar unemployment rate categories have previously been used in the analysis of liver cancer mortality trends, with low and high unemployment rate categories representing approximately 50% below and above the historical average of the national unemployment rate of around 6.00% between 1950 and 2014 or during 1990–2010 [12, 19]. Each of the 3,141 counties in the national mortality database was assigned one of the 4 unemployment categories. Unemployment rates from the 1990 census were used to compute mortality rates by unemployment level for the 1990–1992, 1993–1995, and 1996–1998 periods, whereas unemployment rates from the 2000 census were used in computation of mortality rates for the 1999–2001, 2002–2005, and 2006–2010 periods [12].

Life expectancy estimates, along with associated standard errors and 95% confidence intervals, were derived for six time periods: 1990–1992, 1993–1995, 1996–1998, 1999–2001, 2002–2005, and 2006–2010 [4, 20, 21]. These time periods were used because of the availability and confidential restrictions of county-level mortality data [4, 20, 21]. Age-sex-race-county-specific deaths for these time periods were obtained using the national mortality database [4, 20, 21], whereas age-sex-race-county-specific population estimates for the same time periods served as denominators for computing age-specific mortality rates [4, 21, 22]. Age-sex-race-specific deaths and populations were summed across counties that were grouped into four distinct area unemployment rate categories noted above to produce age-specific mortality rates for each area unemployment group. Life-table estimates were calculated by the standard life-table methodology by converting observed age-specific mortality rates (for 19 age groups: <1, 1–4, 5–9,…, 80–84, and ≥85 years) into life-table probabilities of dying [2, 6, 23, 24]. Infant mortality rate was used to approximate the probability of dying in the first year of life [2, 6].

We examined disparities in life expectancy by stratifying analyses according to race/ethnicity, sex, and area unemployment level for all time periods. Inequalities in life expectancy across sex, race, and time periods were measured by the absolute difference in life expectancy between unemployment categories. Absolute differences in life expectancy in 2006–2010 between low and high unemployment areas were decomposed additively by age groups and into major underlying causes of death using Arriaga's and the standard life-table decomposition methods [24–26]. Other decomposition methods, including those proposed by Pollard, Vaupel and Romo, and Beltrán-Sánchez et al., and the cause-deletion method produce very similar results [27–29]. The Arriaga method is conceptually simple and easy to interpret and has been widely used to decompose life expectancy differences in the United States and elsewhere [2, 24–26, 30]. Life-table survival function was used to analyze trends in survivorship by age, race, and unemployment level. Life expectancy analyses were supplemented by computing all-cause and cause-specific mortality rates in 1990–1992 and 2006–2010 for low and high unemployment areas.

## 3. Results

**Figure 1:**
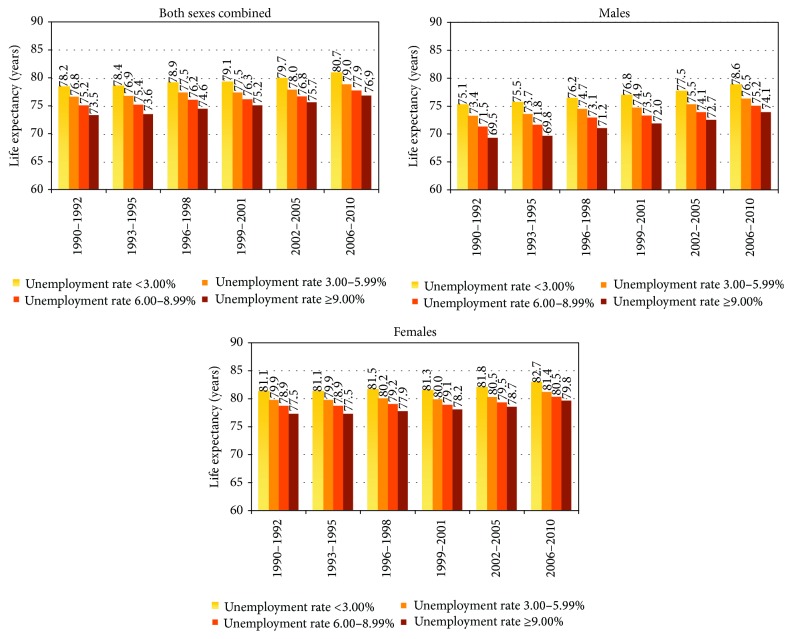
Life expectancy at birth by sex and area unemployment level, United States, 1990–2010.

Life expectancy was inversely related to unemployment levels in all time periods (Table 1 and Figure 1). In 2006–2010, those in areas with high unemployment rates had a life expectancy of 76.9 years, compared with 80.7 years for those in low unemployment areas. Life expectancy varied substantially by time period, sex, and unemployment level, ranging from 69.5 years for men in high unemployment areas in 1990–1992 to 82.7 years for women in low unemployment areas in 2006–2010 (Table 1 and Figure 1). The gradient in life expectancy was steeper for males than for females in all time periods. Disparities in life expectancy by unemployment level persisted over time. In 1990–1992, life expectancy was 4.7 years shorter in high unemployment than in low unemployment areas (73.5 versus 78.2 years). The life expectancy difference between low and high unemployment areas was 4.7 years during 1993–1995, 4.3 years during 1996–1998, 3.9 years during 1999–2001, and 4.0 years during 2002–2005. In 2006–2010, the life expectancy difference between the lowest and highest unemployment areas had decreased to 3.8 years (80.7 versus 76.9 years). The gap in life expectancy between low and high unemployment areas narrowed from 5.6 years in 1990–1992 to 4.5 years in 2006–2010 for males and from 3.6 years in 1990–1992 to 2.9 years in 2006–2010 for females.

Between 1990–1992 and 2006–2010, residents in the highest unemployment areas experienced larger gains in life expectancy than those in the lowest unemployment areas, which contributed to the narrowing gap in life expectancy. For men in the highest unemployment areas, life expectancy increased by 4.6 years between 1990 and 2010, while life expectancy among men in the lowest unemployment areas increased by 3.5 years. The corresponding increases in life expectancy among women in the highest unemployment and lowest unemployment areas were 2.3 and 1.6 years (Table 1).

Unemployment disparities in life expectancy existed for all racial/ethnic groups, particularly among American Indians/Alaska Natives who, on average, lived 8.7 years longer in low unemployment areas than in high unemployment areas during 2006–2010 (Table 1). The association between unemployment and life expectancy was also more marked among black and Hispanic populations who lived 6.3 and 7.7 years longer in low unemployment areas than in high unemployment areas during 2006–2010. When stratified by sex, race, and unemployment level, life expectancy in 2006–2010 ranged from 69.9 years among black men in high unemployment areas to 90.0 among Asian/Pacific Islander women in low unemployment areas (Table 1). Similar group differences existed in 1990–1992, with black men in high unemployment areas having the lowest life expectancy (63.2 years) and Asian/Pacific Islander women in low unemployment areas having the highest life expectancy (87.6 years).

Consistent with the life expectancy trends, compared to low unemployment areas, all-cause mortality in high unemployment areas was 28% higher in 1990–1992 and 24% higher in 2006–2010 (Table 2). Compared to low unemployment areas, all-cause mortality among males aged 25–64 in high unemployment areas was 94% higher in 1990–1992 and 92% higher in 2006–2010. During 1990–2010, relative risk of all-cause mortality among women in high unemployment areas increased from 1.74 to 1.81. Between 1990 and 2010, compared to low unemployment areas, relative risk of mortality in high unemployment areas increased from 1.27 to 1.38 for cardiovascular diseases (CVD), from 1.07 to 1.20 for colorectal cancer, from 1.33 to 1.57 for liver cancer, from 1.50 to 1.70 for stomach cancer, from 1.03 to 1.24 for prostate cancer, from 0.98 to 1.11 for breast cancer, from 1.64 to 1.75 for diabetes, from 1.38 to 1.60 for infectious diseases, from 3.36 to 10.3 for HIV/AIDS, and from 1.13 to 1.41 for pneumonia and influenza. Between 1990 and 2010, relative risk of mortality in high unemployment areas decreased from 1.28 to 1.12 for lung cancer, from 1.50 to 1.30 for unintentional injuries, from 1.38 to 1.19 for kidney diseases, from 2.34 to 1.97 for liver cirrhosis, from 1.17 to 1.12 for COPD, from 5.46 to 5.19 for homicide, and from 1.22 to 0.97 for suicide (Table 2).

**Figure 2:**
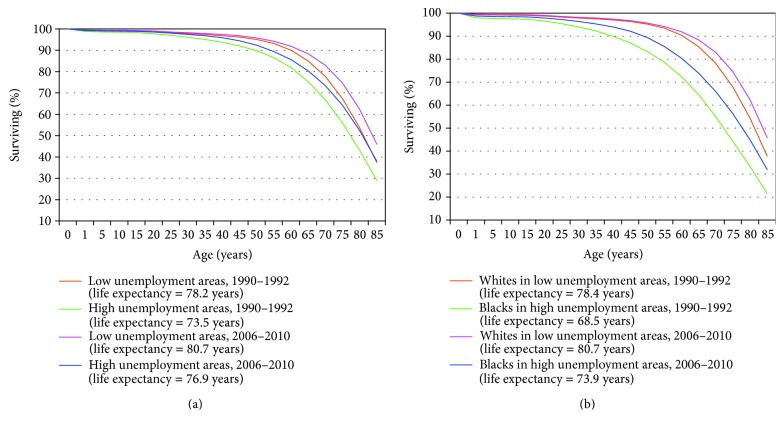
Survivorship by age, race, and unemployment level, United States, 1990–2010.

Although life expectancy increased at all ages between 1990 and 2010, marked disparities by unemployment level remained. In 2006–2010, those aged 25 in low unemployment areas were, on average, expected to live 56.7 additional years, 3.4 years longer than their counterparts in high unemployment areas (53.3 years) (data not shown). Age-specific survivor functions for race and unemployment level indicate that populations in high unemployment areas in 2006–2010 had worse survival experiences than those in low unemployment areas in 1990–1992 and that the black population in high unemployment areas in 2006–2010 experienced substantially lower survival probabilities than those enjoyed by the white population in low unemployment areas two decades earlier (Figure 2).

**Figure 3:**
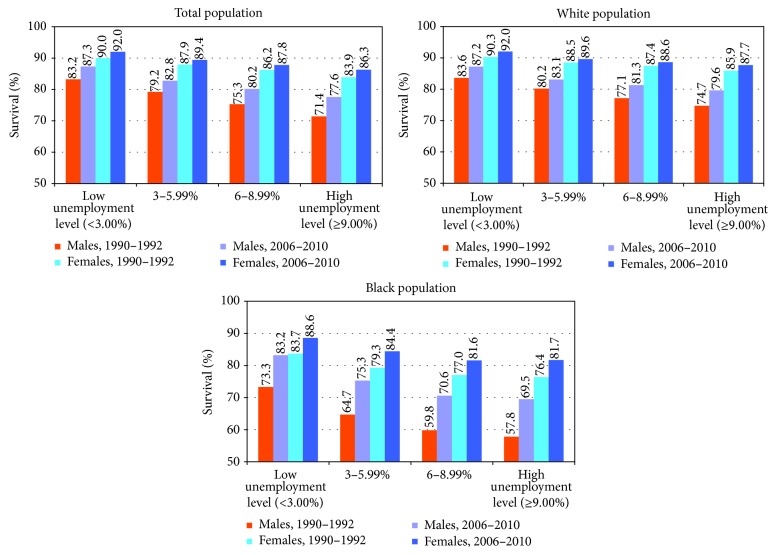
Conditional probability of survival between ages of 25 and 64 years by unemployment level, United States, 1990–2010.

The association between unemployment and the conditional probability of survival between ages of 25 and 64 years is shown for white and black populations in Figure 3. The association between unemployment and survival rates was stronger for men than for women and for blacks than for whites. In 2006–2010, black men in high unemployment areas had a survival rate of 69.5% in the 25–64 age group, almost 14 percentage-points lower than their counterparts from low unemployment areas. In 2006–2010, the population of white women in high unemployment areas had a survival rate of 87.7%, 4 percentage-points lower than their counterparts from low unemployment areas.

Excess mortality under age of 25 in high unemployment areas contributed to 0.40 years or 10.6% of the life expectancy gap of 3.8 years in 2006–2010 (Table 3). Nearly half (45.2%) of the overall life expectancy gap can be attributed to excess mortality among those aged 45–64 in high unemployment areas. Excess mortality among those aged ≥65 in high unemployment areas accounted for 25.1% of the life expectancy gap in women and 20.9% in men. Mortality in the <25 and 25–44 age groups accounted for a larger share of the life expectancy gap in men than in women. Cardiovascular diseases (37.0%), cancer (12.1%), homicide (8.0%), unintentional injuries (8.3%), diabetes (5.9%), HIV/AIDS (4.8%), other infectious diseases (4.6%), liver cirrhosis (3.2%), pneumonia/influenza (2.9%), COPD (2.7%), and kidney diseases (1.6%) accounted for most of the area unemployment gap in overall life expectancy (Table 3). Because of lower suicide and Alzheimer's disease mortality in high unemployment areas, the contribution of suicide and Alzheimer's disease to the life expectancy gap was negative.

Cardiovascular diseases, all cancers, diabetes, HIV/AIDS and other infectious diseases, pneumonia/influenza, and kidney diseases accounted for a larger proportion of the life expectancy gap in women, while homicide, unintentional injuries, liver cirrhosis, COPD, and lung cancer accounted for a greater percentage of the life expectancy gap in men.

## 4. Discussion

Life expectancy in the USA has risen consistently since 1990, increasing from 75.4 years in 1990 to 78.8 years in 2013 [4, 5]. Although life expectancy also increased for population groups in all area unemployment categories between 1990 and 2010, substantial disparities in US life expectancy persisted, with both men and women in high unemployment areas continuing to have 4.5 and 2.9 years shorter life expectancies compared to their counterparts in low unemployment areas. Racial disparities in life expectancy were very marked in both high and low unemployment areas, with black men in high unemployment areas in particular having 5.2 and 13.1 years shorter life expectancies compared to their white and Asian/Pacific Islander counterparts, respectively. When race/ethnicity, sex, and unemployment level are jointly considered, the inequalities are found to be even more dramatic. Black men in high unemployment areas experienced a 22 years shorter life expectancy compared to Asian/Pacific Islander women in low unemployment areas. Remarkably, the black population in high unemployment areas in 2006–2010 experienced life expectancy and survival probabilities that were lower than those experienced by the white population two decades earlier. During the prime working ages, black men in high unemployment areas have 10 percentage-points lower survival rates compared to white men in high unemployment areas and 18 percentage-points lower survival rates compared to white men in low unemployment areas.

With inequalities in mortality from several major causes of death on the rise or continuing to persist, area unemployment disparities in US life expectancy are expected to remain substantial for the foreseeable future [2, 6, 12]. Existence of such marked disparities in US life expectancy by unemployment level and race/ethnicity runs counter to the goals of the national health initiative that calls for elimination of health inequalities by 2020 [1].

Our decomposition analysis showed that CVD, cancer, homicide, unintentional injuries, and diabetes accounted for 71.3% of the overall unemployment gap in life expectancy. Interestingly, area unemployment disparities in mortality from CVD and diabetes have widened over time. Area-level unemployment disparities in life expectancy are compatible with recent studies that show a difference of 4.5 years in life expectancy between the least and most deprived areas and a difference of three to six years between low- and high-poverty areas of the United States [2, 6]. Our study findings are consistent with a recent UK study that showed marked declines in life expectancy in relation to increasing unemployment rates in 324 local authorities of England during 1998–2007 [31].

Unemployment is strongly linked to other deprivation measures such as income inequality and poverty rates in the United States. According to the 2000 US census data, correlations of county unemployment rates with income inequality and poverty rate were 0.56 and 0.83, respectively [22, 32]. Indeed, high unemployment areas in the United States have a five times higher level of income disparity compared to low unemployment areas [22, 32]. Although measures of deprivation and socioeconomic inequality vary across countries, international comparisons can provide important insights into how societal contexts and differences in labor market institutions, welfare structures, and economic policies influence the magnitude of employment-related health inequalities in specific countries [9, 10]. Unemployment disparities in US life expectancy and mortality reported here are mostly consistent with patterns observed for the other industrialized countries [10]. In most studies, the magnitude of the association between unemployment and all-cause mortality is generally higher for men than for women, a finding consistent with our study [10]. A systemic review of studies conducted in the USA, Europe, and a number of other industrialized nations found the average relative risk of all-cause mortality associated with unemployment to be 1.63, which tended to be greater for those in their early and middle careers compared to older workers [10]. Our ecological study showed a nearly twofold higher risk of all-cause mortality among working age men and women in high unemployment areas as compared with a relative risk of 1.24 in the general population.

Consistent with the US pattern, life expectancy in the UK, particularly England and Scotland, has consistently been lower in areas with higher deprivation levels, and these inequalities in life expectancy have either persisted or widened over time [15, 16, 31, 33–36]. However, studies have shown substantially larger health and life expectancy inequalities in the United States than in many other industrialized nations [6, 33, 37]. An analysis of variations in life expectancy across wealthy countries showed a strong inverse relationship between income inequality and life expectancy in selected OECD countries [37]. The existence of larger health inequalities in the US is not surprising, given that income inequality in the United States, whether measured by the Gini coefficient or by the ratio of the average income among the richest 10% to that of the poorest 10% of the population, is among the highest in the OECD countries, exceeded only by Mexico and Chile [38].

A study comparing adverse health effects of unemployment in the United States and Germany found that unemployed Americans, especially those with low education or not receiving unemployment benefits, had the poorest health outcomes [9]. The unemployed had 2.4 times higher risk of mortality in the United States, compared with 1.4 times higher risk in Germany [9]. Consistent with our study, higher unemployment levels were associated with increased risk of mortality in England [39]. A recent study showed higher risks of all-cause mortality and mortality from cardiovascular diseases, all cancers combined and lung cancer, respiratory diseases, and injuries among unemployed Canadian men and women, a finding consistent with the US pattern [40]. A prospective study of 49,321 men in Sweden found a nearly twofold higher risk of all-cause mortality among those experiencing 90 days or more of unemployment compared with the unemployed [41]. In this Swedish study, unemployment in men was also significantly associated with increased risks of mortality from cardiovascular diseases, violent deaths, and suicide [41]. Another study based on the Swedish Twin Registry found an increased risk of premature death and suicide associated with unemployment [42].

Unemployment level inequalities in life expectancy shown here are consistent with disparities in other health measures [9–17]. Residents of higher unemployment areas report significantly higher prevalence of self-assessed fair/poor health, psychological distress, disability, functional limitation, injuries, hypertension, heart disease, diabetes, and other chronic conditions compared to those in low unemployment areas [9–17]. It is not clear whether employment-related disparities in these health indicators have also decreased over time in a pattern similar to those observed for life expectancy and all-cause mortality. Reasons for the narrowing area-level unemployment gap in life expectancy are not entirely clear as trends in life expectancy by other deprivation measures indicate widening health disparities [2, 6, 8]. It is conceivable that socioeconomic and material living conditions of people in high unemployment areas did not worsen relative to those in low unemployment areas during the study period. It is possible that the recent economic downturn, perhaps the worst financial crisis to hit the United States for several decades, affected the health and well-being of both the employed and the unemployed, with the employed and middle-income groups losing their relative health and social advantage during the difficult economic conditions that prevailed during 2006–2010 [18]. One example of this might be the rise (6.4%) in suicide mortality among those in low unemployment areas during 1990–2010 in contrast to individuals in high unemployment areas who experienced a 15.3% drop in their suicide mortality during the same time period (Table 2). Changes in population composition could also partly account for the temporal unemployment disparities in life expectancy. However, the population composition changed a little across the unemployment categories during the two past decades [12]. Trends in unemployment differentials in behavioral risk factors such as smoking, obesity, and physical inactivity also need to be examined to determine their possible contribution to the narrowing gap in life expectancy.

Area unemployment inequalities in life expectancy and mortality may partly reflect inequalities in behavioral and healthcare factors. Unemployment is associated with higher prevalence of smoking, obesity, physical inactivity, and heavy alcohol consumption, as well as lower access to health services in the United States [10, 12, 17]. During 2000–2003, current smoking prevalence among US adults living in high unemployment areas was 23%, compared with 19% among those in low unemployment areas [43]. During 2006–2008, obesity prevalence was 28% among US adults in high unemployment areas, compared with 25% among those in low unemployment areas [43, 44]. Those living in high unemployment areas were also slightly more likely to be physically inactive than those in low unemployment areas [43, 44]. About 9.4% of adults in high unemployment areas during 2006–2008 had diabetes, compared with 7.6% of those in low unemployment areas [43, 44]. Approximately 26.3% of those aged 18–64 years in high unemployment areas lacked health insurance in 2009, compared with 14.2% in low unemployment areas [22, 32].

The unemployment rate is considered an indicator of a community's socioeconomic deprivation as well as its social disintegration or disorganization [6, 15]. Persistent area-level unemployment inequalities in life expectancy shown here may reflect continuing inequalities between high and low unemployment areas in social-structural factors such as material living conditions, social integration, and allocation of other valued social resources (e.g., spending on public safety, social and welfare services, education, affordable housing, job creation, job training, and skills development) [2, 6, 16]. During 2005–2009, median family income in high unemployment areas of the United States was $47,069, compared with $68,64 in low unemployment areas [22, 32]. In 2005–2009, 20.1% of residents in high unemployment areas had a college degree, compared with 29.1% of those in low unemployment areas [22, 32]. In 2005–2009, the poverty rate was 22.7% in high unemployment areas, twice as high as the poverty rate of 11.5% in low unemployment areas [22, 32]. According to the 2000 census, 54% of US counties with high unemployment rates had poor/distressed housing, compared with only 4.4% of counties with low unemployment rates [32]. Violent crime rates are higher in areas with higher unemployment rates. In 2005, the violent crime rate was 781 per 100,000 population in high unemployment areas, compared with 392 in low unemployment areas [32].

Our study has limitations. Life expectancy estimates for Hispanics, Asian/Pacific Islanders, and American Indians/Alaska Natives should be interpreted with caution as vital-statistics-based mortality rates for these groups may be underestimated by 5%, 7%, and 30%, respectively [4, 45]. Life expectancy and mortality estimates for these racial/ethnic groups are often included in national reports and have been published previously [2–4, 46]. Life expectancy estimates for American Indians/Alaska Natives may be underestimated particularly in urban, more affluent counties which have lower unemployment rates than more deprived rural counties [2]. However, compatible with the vital-statistics data, lower all-cause, CVD, and cancer mortality risks among Hispanics and Asian/Pacific Islanders have been found in the National Longitudinal Mortality Study (NLMS) in which race/ethnicity of the respondents whose mortality/survival status is prospectively determined is self-reported [7]. Mortality risks among American Indians/Alaska Natives in the NLMS are estimated to be similar to or lower than those for whites [7]. Secondly, unemployment rates at the neighborhood- or census-tract level could vary greatly within a given county [12, 22]. Unfortunately, census-tract geocodes are not available in the national mortality database [2, 4, 12, 20]. Given the compositional heterogeneity of counties, the association between area unemployment and life expectancy reported here is likely to be underestimated among all sex and race groups [6, 12]. Thirdly, our unemployment measure did not take into account the number of discouraged workers, which would likely have resulted in an underestimation of the full magnitude of the association between unemployment and life expectancy [9, 11]. Lastly, our analysis was based on aggregate-level population data; drawing inferences about individual-level association between unemployment and life expectancy can lead to ecological bias [6, 26].

Improvements in socioeconomic conditions may lead to further increases in life expectancy and reduced mortality rates in areas with high unemployment rates, as they have remained disadvantaged over the past several decades in terms of socioeconomic conditions, economic and educational opportunities, provision of health services, access to essential goods and services, and transportation [2, 6, 22, 32]. Social policies with greater welfare support and social protection for the unemployed can also help reduce the adverse health effects of unemployment [9–12]. Other policy strategies to reduce the health consequences of unemployment may include reducing levels of long-term unemployment and inactivity; integration of employment and skill services; investment in job training and skills development, particularly in high unemployment areas; increasing unemployment benefits and pensions; creating more favorable work environments by reducing job insecurity and work-related stress and increasing autonomy, control, and decision-making in the workplace; raising the minimum wage; and improving incentives to work by providing greater income support through tax credits [9–11, 15, 16, 18]. Behavioral and social policy interventions such as smoking reduction, antiobesity measures, and improved healthcare access have the potential to reduce health and life expectancy inequalities between low and high unemployment areas [2, 6]. However, reducing inequalities in educational opportunities and achievement, poverty, housing, transportation, and labor market opportunities, the underlying social determinants of population health inequalities and which give rise to area inequalities in health-risk behaviors and access to healthcare services, could be an effective policy goal for reducing the area unemployment gap in life expectancy [2, 6, 15, 16].

## 5. Conclusions

Our study shows that life expectancy is inversely related to area unemployment levels. While life expectancy increased for all population groups in the United States, unemployment disparities in life expectancy persisted over time. In 1990–1992, life expectancy was 4.7 years shorter in the highest unemployment areas than in the lowest unemployment areas. In 2006–2010, life expectancy in the highest unemployment areas was 76.9 years, 3.8 years shorter than the life expectancy of 80.7 years in the lowest unemployment areas. Unemployment inequalities in life expectancy were greater for men than for women. Racial disparities in life expectancy were very marked in both high and low unemployment areas, with black men in high unemployment areas in particular having 5.2 and 13.1 years shorter life expectancies than their white and Asian/Pacific Islander counterparts, respectively. Cardiovascular diseases, cancer, homicide, unintentional injuries, and diabetes explained 71.3% of the gap in life expectancy between low and high unemployment areas. Cardiovascular diseases, all cancers, diabetes, HIV/AIDS and other infectious diseases, pneumonia/influenza, and kidney diseases accounted for a larger proportion of the life expectancy gap in women, while homicide, unintentional injuries, liver cirrhosis, COPD, and lung cancer accounted for a greater percentage of the life expectancy gap in men. Unemployment disparities in life expectancy narrowed between 1990 and 2010 as the highest unemployment areas experienced larger gains in life expectancy than the lowest unemployment areas. Area unemployment inequalities in life expectancy and mortality most likely reflect inequalities in material and social conditions as well as those in behavioral and healthcare factors. Social policies aimed at reducing disparities in the broader social determinants such as education, poverty, housing, transportation, and labor market opportunities, along with public health measures that target reductions in smoking, obesity, and alcohol consumption and improvements in access to health services, have the potential to reduce health and life expectancy inequalities between low and high unemployment areas.

## Figures and Tables

**Table 1:** Life expectancy at birth (in years) by area unemployment level, race/ethnicity, and sex, United States, 1990–1992 and 2006–2010.

	2006–2010						1990–1992						Difference, 1990–2010					
	Both sexes		Males		Females		Both sexes		Males		Females		Both sexes		Males		Females	
	LE	95% CI	LE	95% CI	LE	95% CI	LE	95% CI	LE	95% CI	LE	95% CI						
Unemployment level, total US population																		
<3.00%	80.7	80.7–80.8	78.6	78.6–78.7	82.7	82.7–82.8	78.2	78.1–78.2	75.1	75.0–75.2	81.1	81.0–81.2	2.5	2.4–2.6	3.5	3.4–3.6	1.6	1.5–1.7
3–5.99%	79.0	79.0–79.0	76.5	76.5–76.5	81.4	81.4–81.4	76.8	76.8–76.8	73.4	73.4–73.5	79.9	79.9–80.0	2.2	2.2–2.2	3.1	3.1–3.1	1.5	1.5–1.5
6–8.99%	77.9	77.0–77.9	75.2	75.2–75.2	80.5	80.5–80.5	75.2	75.2–75.2	71.5	71.5–71.5	78.9	78.8–78.9	2.7	2.7–2.7	3.7	3.7–3.7	1.6	1.5–1.5
≥9.00%	76.9	76.8–77.0	74.1	74.0–74.1	79.8	79.8–79.9	73.5	73.4–73.5	69.5	69.4–69.5	77.5	77.4–77.5	3.4	3.4–3.4	4.6	4.5–4.7	2.3	2.2–2.4
Low−high unemployment areas^1^	3.8	3.8–3.8	4.5	4.4–4.6	2.9	2.8–3.0	4.7	4.6–4.8	5.6	5.5–5.7	3.6	3.5–3.7						
Unemployment level, whites																		
<3.00%	80.7	80.6–80.7	78.5	78.5–78.6	82.7	82.6–82.7	78.4	78.3–78.4	75.3	75.2–75.4	81.2	81.2–81.3	2.3	2.2–2.4	3.2	3.1–3.3	1.5	1.4–1.6
3–5.99%	79.1	79.1–79.1	76.7	76.7–76.7	81.5	81.5–81.5	77.2	77.2–77.2	73.9	73.9–74.0	80.3	80.3–80.4	1.9	1.9–1.9	2.8	2.8–2.8	1.2	1.2–1.2
6–8.99%	78.3	78.3–78.3	75.8	75.8–75.8	80.9	80.8–80.9	76.0	76.0–76.0	72.4	72.4–72.5	79.5	79.5–79.6	2.3	2.3–2.3	3.4	3.4–3.4	1.4	1.4–1.4
≥9.00%	77.8	77.7–77.8	75.1	75.1–75.2	80.4	80.4–80.5	74.8	74.8–74.9	71.1	71.1–71.2	78.6	78.5–78.6	3.0	2.9–3.1	4.0	3.9–4.1	1.8	1.7–1.9
Low−high unemployment areas	2.9	2.9–3.0	3.4	3.3–3.5	2.3	2.2–2.4	3.6	3.5–3.7	4.2	4.1–4.3	2.6	2.5–2.7						
Unemployment level, blacks																		
<3.00%	78.6	78.5–78.8	76.2	76.0–76.5	80.7	80.5–82.9	73.5	73.2–73.9	69.7	69.3–70.2	77.2	76.7–77.6	5.1	4.7–5.5	6.5	6.0–7.0	3.5	3.0–4.0
3–5.99%	75.5	75.5–75.5	72.3	72.3–72.4	78.4	78.3–78.4	70.8	70.7–70.9	66.5	66.4–66.6	74.9	74.8–75.0	4.7	4.6–4.8	5.8	5.7–5.9	3.5	3.4–3.6
6–8.99%	73.8	73.8–73.8	70.3	70.2–70.3	77.0	77.0–77.1	69.1	69.1–69.2	64.2	64.2–64.3	73.9	73.9–74.0	4.7	4.6–4.8	6.1	6.0–6.2	3.1	3.0–3.2
≥9.00%	73.9	73.9–74.0	69.9	69.9–70.0	77.5	77.4–77.6	68.5	68.5–68.6	63.2	63.1–63.4	73.6	73.5–73.7	5.4	5.3–5.5	6.7	6.6–6.8	3.9	3.8–4.0
Low−high unemployment areas	4.7	4.5–4.9	6.3	6.0–6.6	3.2	3.0–3.4	5.0	4.7–5.3	6.5	6.1–7.1	3.6	3.1–4.1						
Unemployment level, American Indians, and Alaska Natives																		
<3.00%	85.3	84.5–86.0	83.0	82.0–84.1	87.2	86.2–88.2	a	a	a	a	a	a						
3–5.99%	82.6	82.5–82.8	80.4	80.1–80.6	84.7	84.6–84.9	80.6	80.3–81.0	76.7	76.2–77.2	84.1	83.6–84.6	2.0	1.6–2.4	3.7	3.2–4.2	0.6	0.1–1.1
6–8.99%	81.3	81.2–81.5	78.9	78.7–79.1	83.5	83.3–83.7	80.4	80.2–80.7	76.1	75.7–76.4	84.6	84.2–85.0	0.9	0.6–1.2	2.8	2.4–3.2	−1.1	−1.5, −0.7
≥9.00%	76.6	76.4–76.8	73.2	73.0–73.5	80.0	79.7–80.2	72.9	72.6–73.2	68.4	68.0–68.8	77.6	77.2–78.0	3.7	3.4–4.0	4.8	4.3–5.3	2.4	1.9–2.9
Low−high unemployment areas	8.7	7.9–9.5	9.8	8.7–10.9	7.2	6.2–8.2	7.7	7.2–8.2	8.3	7.7–8.9	6.5	5.9–7.1						
Unemployment level, Asians and Pacific Islanders																		
<3.00%	88.2	88.0–88.4	86.0	85.7–86.4	90.0	89.7–90.3	86.0	85.4–86.6	84.2	83.2–85.2	87.6	86.8–88.3	2.2	1.6–2.8	1.8	0.8–2.8	2.4	1.6–3.2
3–5.99%	87.1	87.1–87.2	84.9	84.8–84.9	89.0	89.0–89.1	82.4	82.3–82.5	79.6	79.5–79.8	85.3	85.1–85.4	4.7	4.6–4.8	5.3	5.1–5.5	3.7	3.5–3.9
6–8.99%	85.7	85.6–85.7	83.0	82.9–83.0	88.1	88.0–88.2	83.0	82.9–83.1	80.0	79.9–80.2	86.0	85.8–86.1	2.7	2.6–2.8	3.0	2.8–3.2	2.1	1.9–2.3
≥9.00%	85.7	85.6–85.9	83.0	82.8–83.2	88.3	88.1–88.5	82.6	82.3–83.0	79.6	79.2–80.1	86.1	85.6–86.6	3.1	2.7–3.5	3.4	2.9–3.9	2.2	1.7–2.7
Low−high unemployment areas	2.5	2.2–2.8	3.0	2.6–3.4	1.7	1.3–2.1	3.4	2.7–4.1	4.6	3.5–5.7	1.5	0.6–2.4						
Unemployment level, Hispanics																		
<3.00%	86.8	86.6–87.1	85.4	85.0–85.8	88.3	88.0–88.6	84.1	83.6–84.7	80.6	79.7–81.6	86.7	86.0–87.5	2.7	2.1–3.3	4.8	3.8–5.8	1.6	0.8–2.4
3–5.99%	83.0	83.0–83.1	80.4	80.4–80.5	85.5	85.4–85.5	82.0	81.9–82.1	78.2	78.1–78.4	85.8	85.6–85.9	1.0	0.9–1.1	2.2	2.0–2.4	−0.3	−0.5, −0.1
6–8.99%	82.3	82.3–82.4	79.4	79.4–79.5	85.1	85.0–85.1	79.5	79.4–79.5	75.3	75.2–75.4	83.7	83.6–83.7	2.8	2.7–2.9	4.1	4.0–4.2	1.4	1.3–1.5
≥9.00%	80.7	80.7–80.8	77.7	77.6–77.8	83.6	83.5–83.7	77.3	77.2–77.4	73.1	73.0–73.2	81.5	81.3–81.6	3.4	3.3–3.5	4.6	4.4–4.8	2.1	1.9–2.3
Low−high unemployment areas	6.1	5.4–6.8	7.7	7.3–8.1	4.7	4.4–5.0	6.8	6.2–7.4	7.5	6.5–8.5	5.2	4.5–5.9						

Life expectancy estimates for non-Hispanic whites were similar to those for whites. LE = life expectancy. CI = confidence interval. a = insufficient data.

^1^Difference in life expectancy between low unemployment areas (rate < 3.00%) and high unemployment areas (rate ≥ 9.00%).

Source: based on data from the US National Vital Statistics System, 1990–2010 [20, 21].

**Table 2:** All-cause and cause-specific mortality rates^1^ per 100,000 population for low and high unemployment areas, United States, 1990–1992 and 2006–2010.

	2006–2010						1990–1992					
	Low unemployment area (<3%)		High unemployment area (≥9%)		High/low unemployment		Low unemployment area (<3%)		High unemployment area (≥9%)		High/low unemployment	
	Rate	SE	Rate	SE	Rate ratio	95% CI	Rate	SE	Rate	SE	Rate ratio	95% CI
All causes of death	671.10	1.06	834.14	0.87	**1.24**	1.24–1.25	807.43	2.24	1031.03	1.16	**1.28**	1.27–1.28
Males 25–64 (working age)	283.52	1.21	544.27	1.35	**1.92**	1.90–1.94	371.24	2.86	721.91	1.99	**1.94**	1.91–1.98
Females 25–64 (working age)	172.70	0.93	313.06	1.00	**1.81**	1.79–1.83	211.66	2.14	368.14	1.36	**1.74**	1.70–1.78
Cardiovascular diseases (CVD)	208.59	0.59	287.47	0.51	**1.38**	1.37–1.39	347.76	1.49	443.34	0.77	**1.27**	1.26–1.29
Diseases of heart	153.65	0.51	226.16	0.45	**1.47**	1.46–1.48	267.23	1.30	357.02	0.69	**1.34**	1.32–1.35
Hypertension	6.05	0.10	10.57	0.10	**1.75**	1.68–1.81	3.02	0.14	4.90	0.08	**1.62**	1.47–1.78
Stroke	39.01	0.26	42.24	0.20	**1.08**	1.07–1.10	59.79	0.62	63.51	0.29	**1.06**	1.07–1.10
All malignant cancers	160.46	0.52	181.34	0.41	**1.13**	1.12–1.14	196.85	1.10	225.62	0.54	**1.15**	1.13–1.16
Oral cavity and pharynx	1.94	0.06	2.71	0.05	**1.40**	1.30–1.50	2.64	0.13	3.79	0.07	**1.44**	1.29–1.58
Digestive system	38.35	0.25	46.88	0.21	**1.22**	1.20–1.24	46.46	0.53	54.47	0.27	**1.17**	1.14–1.20
Esophagus	4.10	0.08	4.04	0.06	0.99	0.94–1.03	3.53	0.15	4.64	0.08	**1.31**	1.20–1.43
Stomach	2.73	0.07	4.63	0.07	**1.70**	1.60–1.79	4.64	0.17	6.94	0.09	**1.50**	1.38–1.61
Colorectal	14.88	0.16	17.89	0.13	**1.20**	1.17–1.23	23.04	0.38	24.56	0.18	**1.07**	1.03–1.10
Liver & intrahepatic bile duct	4.34	0.08	6.83	0.08	**1.57**	1.51–1.64	3.19	0.14	4.24	0.07	**1.33**	1.21–1.45
Pancreatic	10.14	0.13	11.01	0.10	**1.09**	1.05–1.12	9.74	0.24	11.21	0.12	**1.15**	1.09–1.21
Respiratory system	44.38	0.27	50.33	0.21	**1.13**	1.12–1.15	50.91	0.55	66.00	0.29	**1.30**	1.27–1.33
Larynx	0.80	0.04	1.50	0.04	**1.88**	1.67–2.08	1.07	0.08	2.04	0.05	**1.91**	1.61–2.20
Lung and bronchus	43.34	0.27	48.55	0.21	**1.12**	1.10–1.14	49.47	0.55	63.45	0.28	**1.28**	1.25–1.31
Melanoma of the skin	3.02	0.07	2.18	0.04	**0.72**	0.68–0.76	2.67	0.13	2.23	0.05	**0.84**	0.75–0.92
Breast	21.28	0.25	23.59	0.20	**1.11**	1.08–1.14	32.48	0.59	31.77	0.27	0.98	0.94–1.02
Cervical	1.54	0.07	3.37	0.08	**2.19**	1.97–2.41	2.52	0.17	4.94	0.11	**1.96**	1.69–2.23
Uterine	3.86	0.11	4.97	0.09	**1.29**	1.20–1.37	4.31	0.21	4.53	0.10	1.05	0.94–1.16
Prostate	21.44	0.31	26.55	0.25	**1.24**	1.20–1.28	39.23	0.85	40.57	0.39	1.03	0.99–1.08
Bladder	4.20	0.08	4.06	0.06	0.97	0.92–1.01	4.31	0.16	4.31	0.07	1.00	0.92–1.08
Kidney	3.77	0.08	4.09	0.06	**1.08**	1.03–1.14	4.20	0.16	4.51	0.08	1.07	0.99–1.16
Brain	4.45	0.08	3.64	0.06	**0.82**	0.78–0.86	5.12	0.17	4.50	0.08	**0.88**	0.81–0.94
Non-Hodgkin's lymphoma	6.19	0.10	6.12	0.07	0.99	0.95–1.03	8.14	0.22	7.71	0.10	0.95	0.89–1.14
Myeloma	3.30	0.07	3.48	0.06	1.05	1.00–1.11	3.55	0.15	3.93	0.07	**1.11**	1.01–1.21
Leukemia	7.01	0.11	6.79	0.08	0.97	0.93–1.01	7.88	0.22	7.98	0.10	1.01	0.95–1.07
Infectious diseases without HIV	14.18	0.15	22.73	0.14	**1.60**	1.56–1.64	12.55	0.28	17.32	0.15	**1.38**	1.32–1.44
Tuberculosis	0.10	0.01	0.33	0.02	**3.30**	2.54–4.06	0.31	0.04	1.39	0.04	**4.48**	3.32–5.65
Septicemia	9.34	0.13	14.50	0.12	**1.55**	1.71–1.79	9.13	0.24	11.07	0.12	**1.21**	1.14–1.28
HIV/AIDS	0.81	0.03	8.31	0.09	**10.26**	9.48–11.04	4.73	0.16	15.89	0.14	**3.36**	3.13–3.59
Diabetes mellitus	16.62	0.17	29.16	0.16	**1.75**	1.71–1.79	15.86	0.31	25.98	0.18	**1.64**	1.57–1.70
Alzheimer's disease	24.72	0.21	19.75	0.14	**0.80**	0.78–0.82	6.96	0.21	5.00	0.08	**0.72**	0.67–0.77
Pneumonia and influenza	14.86	0.16	20.89	0.14	**1.41**	1.37–1.44	34.17	0.47	38.54	0.23	**1.13**	1.09–1.16
COPD	36.96	0.25	41.22	0.20	**1.12**	1.10–1.13	33.05	0.45	38.68	0.22	**1.17**	1.14–1.20
Chronic liver disease & cirrhosis	5.80	0.09	11.41	0.10	**1.97**	1.90–2.04	5.84	0.19	13.68	0.14	**2.34**	2.19–2.50
Nephritis/kidney diseases	13.67	0.15	16.28	0.12	**1.19**	1.16–1.22	8.44	0.23	11.66	0.12	**1.38**	1.30–1.46
Unintentional injuries	33.36	0.23	43.29	0.20	**1.30**	1.28–1.32	31.14	0.43	46.62	0.24	**1.50**	1.45–1.54
Suicide	10.75	0.13	10.43	0.10	0.97	0.94–1.00	10.10	0.24	12.32	0.12	**1.22**	1.16–1.28
Homicide	2.10	0.06	10.89	0.10	**5.19**	4.88–5.49	3.27	0.13	17.84	0.15	**5.46**	5.02–5.89

^1^Mortality rates are age-adjusted to the 2000 US population standard. The 2000 census county-level unemployment rates were used to compute mortality rates in 2006–2010, whereas the 1990 census unemployment rates were used to calculate mortality rates in 1990–1992.

**Table 3:** Contribution of specific age groups and leading causes of death to the gap in life expectancy at birth between low and high unemployment areas of the United States, 2006–2010.

Cause of death	Both sexes		Males		Females	
	Absolute contribution (years)	Percentage contribution	Absolute contribution (years)	Percentage contribution	Absolute contribution (years)	Percentage contribution
All ages and causes	3.76	100.0	4.54	100.0	2.91	100.0
Age group (years)						
<25	0.40	10.6	0.51	11.2	0.28	9.6
25–44	0.83	22.1	1.05	23.1	0.59	20.3
45–64	1.70	45.2	2.03	44.7	1.31	45.0
≥65	0.83	22.1	0.95	20.9	0.73	25.1
*Cause of death*						
Cardiovascular diseases (CVD)	1.38	37.0	1.48	32.6	1.27	43.6
Heart disease	1.22	32.7	1.28	28.2	1.16	39.9
Stroke	0.10	2.7	0.13	2.9	0.07	2.4
Hypertension	0.08	2.1	0.08	1.8	0.08	2.7
All cancers combined	0.45	12.1	0.51	11.2	0.40	13.7
Lung cancer	0.11	2.9	0.16	3.5	0.07	2.4
Colorectal cancer	0.06	1.6	0.06	1.3	0.05	1.7
Prostate cancer	0.03	0.8	0.06	1.3		
Breast cancer	0.03	0.8			0.06	2.1
Cervical cancer	0.02	0.5			0.04	1.4
Stomach cancer	0.03	0.8	0.04	0.9	0.03	1.0
Liver & intrahepatic bile duct cancer	0.05	1.3	0.07	1.5	0.03	1.0
Chronic lower respiratory diseases/COPD	0.10	2.7	0.14	3.1	0.07	2.4
Diabetes mellitus	0.22	5.9	0.20	4.4	0.24	8.2
Influenza and pneumonia	0.11	2.9	0.12	2.6	0.11	3.8
Nephritis/kidney diseases	0.06	1.6	0.06	1.3	0.07	2.4
Chronic liver disease and cirrhosis	0.12	3.2	0.17	3.7	0.08	2.7
HIV/AIDS	0.18	4.8	0.16	3.5	0.14	4.8
Infectious/parasitic diseases excluding HIV/AIDS	0.17	4.6	0.19	4.2	0.16	5.5
Alzheimer's disease	−0.06	−1.6	−0.03	−0.7	−0.09	−3.1
Unintentional injuries	0.31	8.3	0.44	9.7	0.16	5.5
Suicide	−0.01	−0.3	−0.01	−0.2	−0.02	−0.7
Homicide	0.30	8.0	0.51	11.2	0.08	2.7
All other causes	0.40	10.7	0.60	13.2	0.24	8.2

Source: derived from 2006–2010 data from the US National Vital Statistics System [20, 21].
